# Deep‐learning‐based renal artery stenosis diagnosis via multimodal fusion

**DOI:** 10.1002/acm2.14298

**Published:** 2024-02-19

**Authors:** Xin Wang, Sheng Cai, Hongyan Wang, Jianchu Li, Yuqing Yang

**Affiliations:** ^1^ Department of Ultrasound, State Key Laboratory of Complex Severe and Rare Diseases Peking Union Medical College Hospital, Chinese Academy of Medical Science and Peking Union Medical College Beijing China; ^2^ State Key Laboratory of Networking and Switching Technology Beijing University of Posts and Telecommunications Beijing China

**Keywords:** color doppler sonography, deep learning, multimodal fusion, renal artery stenosis, renal artery ultrasound

## Abstract

**Purpose:**

Diagnosing Renal artery stenosis (RAS) presents challenges. This research aimed to develop a deep learning model for the computer‐aided diagnosis of RAS, utilizing multimodal fusion technology based on ultrasound scanning images, spectral waveforms, and clinical information.

**Methods:**

A total of 1485 patients received renal artery ultrasonography from Peking Union Medical College Hospital were included and their color doppler sonography (CDS) images were classified according to anatomical site and left‐right orientation. The RAS diagnosis was modeled as a process involving feature extraction and multimodal fusion. Three deep learning (DL) models (ResNeSt, ResNet, and XCiT) were trained on a multimodal dataset consisted of CDS images, spectrum waveform images, and individual basic information. Predicted performance of different models were compared with senior physician and evaluated on a test dataset (*N* = 117 patients) with renal artery angiography results.

**Results:**

Sample sizes of training and validation datasets were 3292 and 169 respectively. On test data (*N* = 676 samples), predicted accuracies of three DL models were more than 80% and the ResNeSt achieved the accuracy 83.49% ± 0.45%, precision 81.89% ± 3.00%, and recall 76.97% ± 3.7%. There was no significant difference between the accuracy of ResNeSt and ResNet (82.84% ± 1.52%), and the ResNeSt was higher than the XCiT (80.71% ± 2.23%, *p* < 0.05). Compared to the gold standard, renal artery angiography, the accuracy of ResNest model was 78.25% ± 1.62%, which was inferior to the senior physician (90.09%). Besides, compared to the multimodal fusion model, the performance of single‐modal model on spectrum waveform images was relatively lower.

**Conclusion:**

The DL multimodal fusion model shows promising results in assisting RAS diagnosis.

## 1 INTRODUCTION

Renal artery stenosis (RAS) is a prevalent secondary cause of hypertension, accounting for approximately 5%−10% of all hypertension cases.^1^, ^2^ RAS can result in renal hypoperfusion, renal dysfunction, cardiac destabilization syndrome, and end‐stage renal disease, all of which have significant healthcare implications and require prompt treatment.^3^, ^4^ Atherosclerosis disease is the primary cause of RAS in the elderly, responsible for about 90% of cases, while vasculitis and renal fibrovascular dysplasia are more common causes in younger patients, particularly young women.

Imaging examinations are crucial for diagnosing RAS, and selective renal artery angiography is considered the gold standard. Nevertheless, due to its invasiveness and high cost, it is not ideal for screening RAS. Currently, color Doppler ultrasound is the preferred method for RAS screening, offering several advantages, including safety, non‐invasiveness, and affordability.^5^

Diagnosing RAS with color doppler ultrasound requires a comprehensive analysis of gray scale, color doppler ultrasound, spectrum, as well as upstream and downstream vascular conditions, which demands skills and a long learning cycle. Operators play a crucial role, and inexperienced physicians are more likely to make misdiagnoses.^5^, ^6^, ^7^ Studies report that color doppler ultrasound has a sensitivity ranging from 54% to 98% and a specificity ranging from 54% to 99% in detecting RAS.^5^, ^6^, ^8^, ^9^

Due to the complexity of RAS diagnosis, a new Computer‐Aided Diagnosis (CAD) technology is needed in clinical practice to objectively and accurately assist in the RAS diagnosis by integrating ultrasound images, hemodynamic parameters, and clinical information from patients. The continuous advancement of artificial intelligence methods, represented by Deep Learning (DL), has promoted the development of CAD technology. In the field of ultrasound, many researchers have trained DL models on ultrasound images to identify lesions in the thyroid,^10^, ^11^ breast,^12^, ^13^ liver.^14^, ^15^ Recently, the CAD technology based on multimodal fusion is adopted in some studies to improve the predictive ability of DL model further, by analyzing multiple types of image data, text data, and numerical data.

DL multimodal fusion technology refers to utilizing suitable DL network structures (such as fully connected networks, CNNs, and BERT) to extract features from different types of data, followed by information fusion to achieve prediction of the target.^16^ In 2014, Suk et al. built a DL model to predict the Alzheimer's disease via fusion of multimodal information from MRI and PET images.^17^ In 2016, Tao et al. designed a model for cervical dysplasia diagnosis by leveraging data from HPV test, Pap test, and cervix images. In 2021, Huang et al. improved the CNN model by combining four types of sonography to assist breast cancer diagnosis.^18^ In 2022, Zhuo et al. proposed a model to detect thyroid cancer lesions based on three modalities (gray‐scale ultrasound, color doppler ultrasound, shear wave elastography).^19^ Based on multimodal fusion, these DL models achieved better performance than models from single‐modal data. Recently, Transformer‐structure‐based models such as ViT, ViLBERT, and ViLT have shown excellent performance in image and text feature extraction and information fusion tasks, promoting the further development of multimodal fusion technology.^20^, ^21^, ^22^

However, for the RAS diagnosis, AI‐based CAD research is still limited. In 2023, Blain et al. analyzed ultrasonography results from 80 renal transplant patients using multiple machine learning methods to evaluate the importance of different measurement parameters in predicting transplant RAS.^23^ To the best of our knowledge, deep‐learning‐based RAS diagnosis model via multimodal fusion has not yet been undertaken.

Renal artery ultrasound diagnosis requires physicians to comprehensively analyze and interpret complex and variable hemodynamic parameters, morphological information, and clinical information, which can be modeled as a process of inputting multimodal data obtained during the ultrasound scanning process, extracting and fusing information, and outputting the degree of vascular stenosis. By combining ultrasound scanning images, spectral waveforms, and clinical information, our study aims to build a new DL multimodal fusion model to assist in RAS diagnosis.

## 2 METHODS

### 2.1 Study design

In this study, we consecutively included patients diagnosed with RAS (reduction of the lumen between 50% and 99%) who underwent renal artery ultrasound examination performed by physicians with no less than 3 years of ultrasound experience at Peking Union Medical College Hospital between August 2017 and December 2019. RAS was diagnosed when the peak systolic velocity (PSV) in the renal artery was ≥ 150 cm/s, or the ratio of PSV in the renal artery to PSV in the aorta (RAR) was ≥ 2.5, or a tardus‐parvus waveform was present.^21^, ^22^, ^23^ Due to the low incidence rate of RAS and the use of ultrasound as a screening method, the number of patients with RAS was lower than those without RAS. To ensure comparability, we randomly selected a comparable number of patients without RAS during the same time period as those with RAS. Two physicians recognized patients with and without RAS based on their ultrasound examination results. Subsequently, the study screened patients based on the completeness of images of the relevant areas, removing those lacking key images of the renal artery region, and utilized the remaining images for the subsequent model training.

This study included 449 patients with RAS and 1036 patients without RAS. Ultrasound images were categorized into four major groups based on anatomical location, namely, Abdominal Aorta (AO), Normal Renal Artery (NRA), RAS, Intrarenal Interlobular Artery (IRA), and basic statistical information is presented in Table 1. The number of images for patients with RAS and those without RAS were comparable, with a total of 1712 and 2425 images, respectively.

**TABLE 1:** Basic characteristics of clinical dataset.

Patient Number	RAS = 449 person		NR = 1036 person	
Orientation	Left	Right	Left	Right
RAS (Image Num)	267	272	0	0
NRA	174	175	842	856
IRA	349	377	654	676
AO	210		313	
Train (Sample Num)	1365		1927	
Val	70		99	
Test	277		399	

Abbreviations: AO, Abdominal Aorta; IRA, Intrarenal Interlobular Artery; NRA, Normal Renal Artery; RAS, Renal Artery Stenosis.

This study was approved by the Institutional Review Board of Peking Union Medical College Hospital (Ethical review number: JS‐3306) and conducted in accordance with the principles of the Declaration of Helsinki.

### 2.2 Data preprocessing

Composite images were created by merging ultrasound scanning images and spectral waveform images from identical vascular areas. Except the AO image, the NRA, RAS, and IRA images were further classified based on their orientation (left or right) in the renal artery.

Next, images from the same side (either left or right) of a patient, specifically from the renal artery, IRA, and AO, were integrated in a specific order to form a multimodal image sample. In practice, the AO images of a given patient were combined with both the left‐sided and right‐sided images.

In order to normalize the input for the proposed models, a proportional scaling method was implemented, filling the residual areas with blank content. Subsequently, each RGB value was divided by 255 to ensure all image RGB values entering the models were confined within the [0,1] range.

Alongside the imaging data, the incorporation of clinical text data from patients was performed. This included normalized age (calculated by subtracting the mean and dividing by the standard deviation), gender (designated as 0 for males and 1 for females), outpatient status (0/1), emergency patient status (0/1), and inpatient status (0/1).

### 2.3 DL multimodal model

The RAS auxiliary diagnostic model constructed in this study is a multimodal DL model. This model is capable of integrating heterogeneous input data, merging the extracted image features from the image feature extraction network portion with clinical information in the fully connected layer. The data undergoes a series of processing steps within the model, which in turn generates predictive output indicating the presence or absence of RAS disease. The aim of this model is to aid and enhance clinical diagnostic procedures.

**FIGURE 1:**
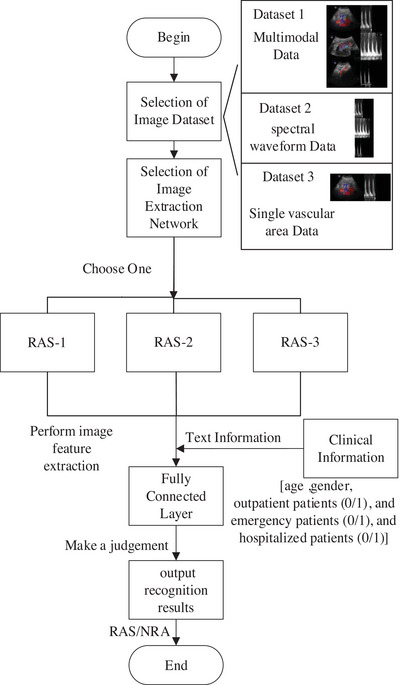
Auxiliary diagnostic structure diagram for RAS disease. RAS, renal artery stenosis.

**FIGURE 2:**
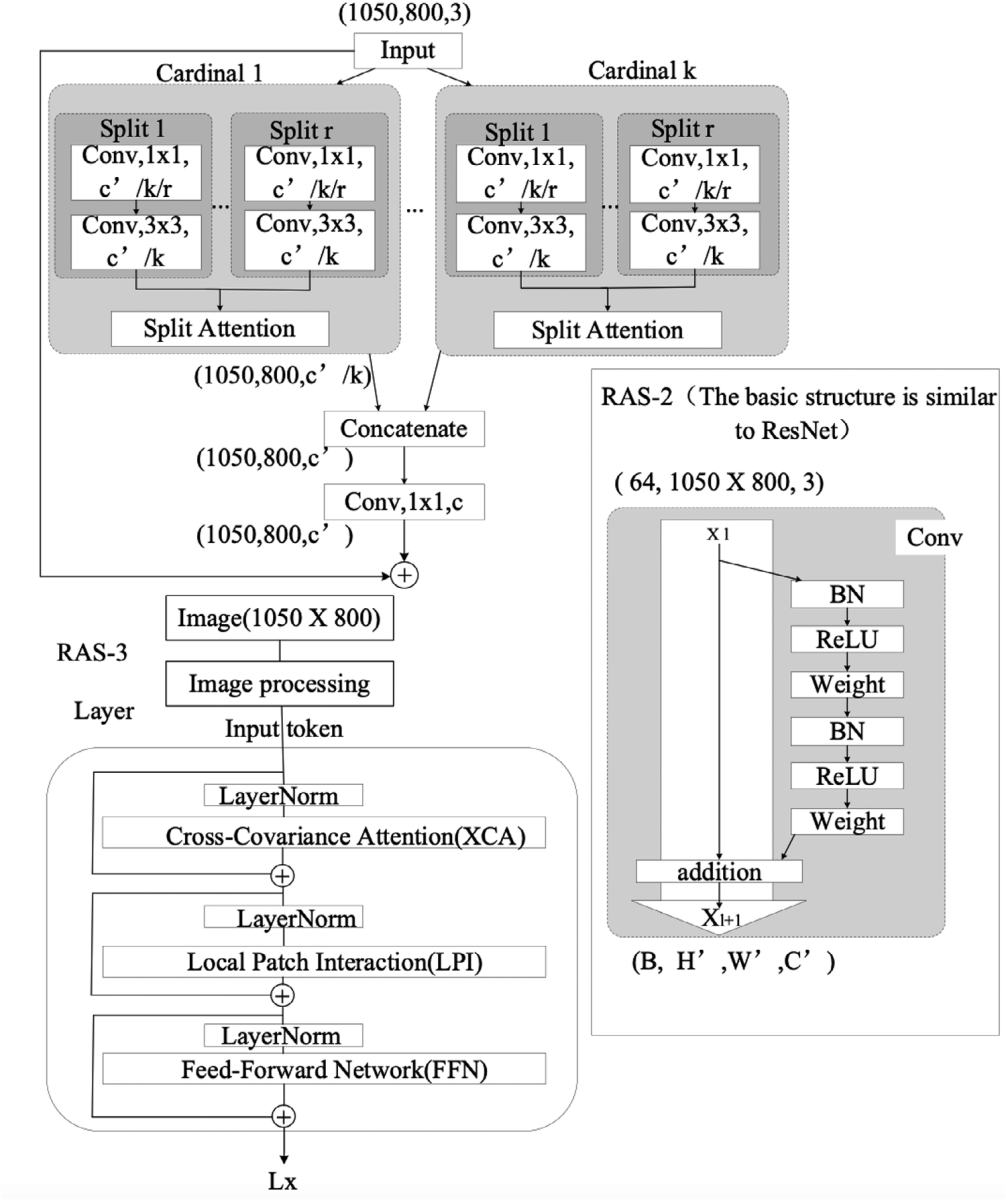
RAS‐1/2/3 simple model structure diagram. RAS, renal artery stenosis.

The construction of our multimodal model is depicted in Figure 1. The high‐performance image feature extraction network we used in this study can better process patient image data to obtain more accurate research results. In this study, three different DL networks, ResNeSt,^24^ ResNet,^25^ and XCiT,^26^ were used as image feature extraction components of our model, and three independent experimental models, namely RAS‐1, RAS‐2 and RAS‐3, were constructed (Figure 2). The distinctive aspect of the RAS‐1 model stems from the “Split‐Attention” characteristic inherent in the ResNeSt network structure. The distinctive aspect of the RAS‐2 model primarily lies in the employment of the ResNetv2 architecture, which incorporates a pre‐activation structure and a specific residual block configuration to optimize network performance and training stability. The RAS‐3 model is unique in that XCiT combines the advantages of convolutional neural networks (CNN) and self‐attention mechanisms, and can capture global correlations in images well by using cross covariance operations.

These networks proficiently extract pertinent information from the patient images, subsequently forwarding this data to a fully connected layer. At the same time, clinical textual data, comprising structured elements such as age, gender, and patient type, are processed through a simple yet effective approach. These processed textual data are then also delivered to the fully connected layer. At this juncture, the image and textual data are fused, contributing to the final auxiliary diagnosis. And the model finally outputs the auxiliary diagnosis result.

### 2.4 Model training and evaluation

**FIGURE 3:**
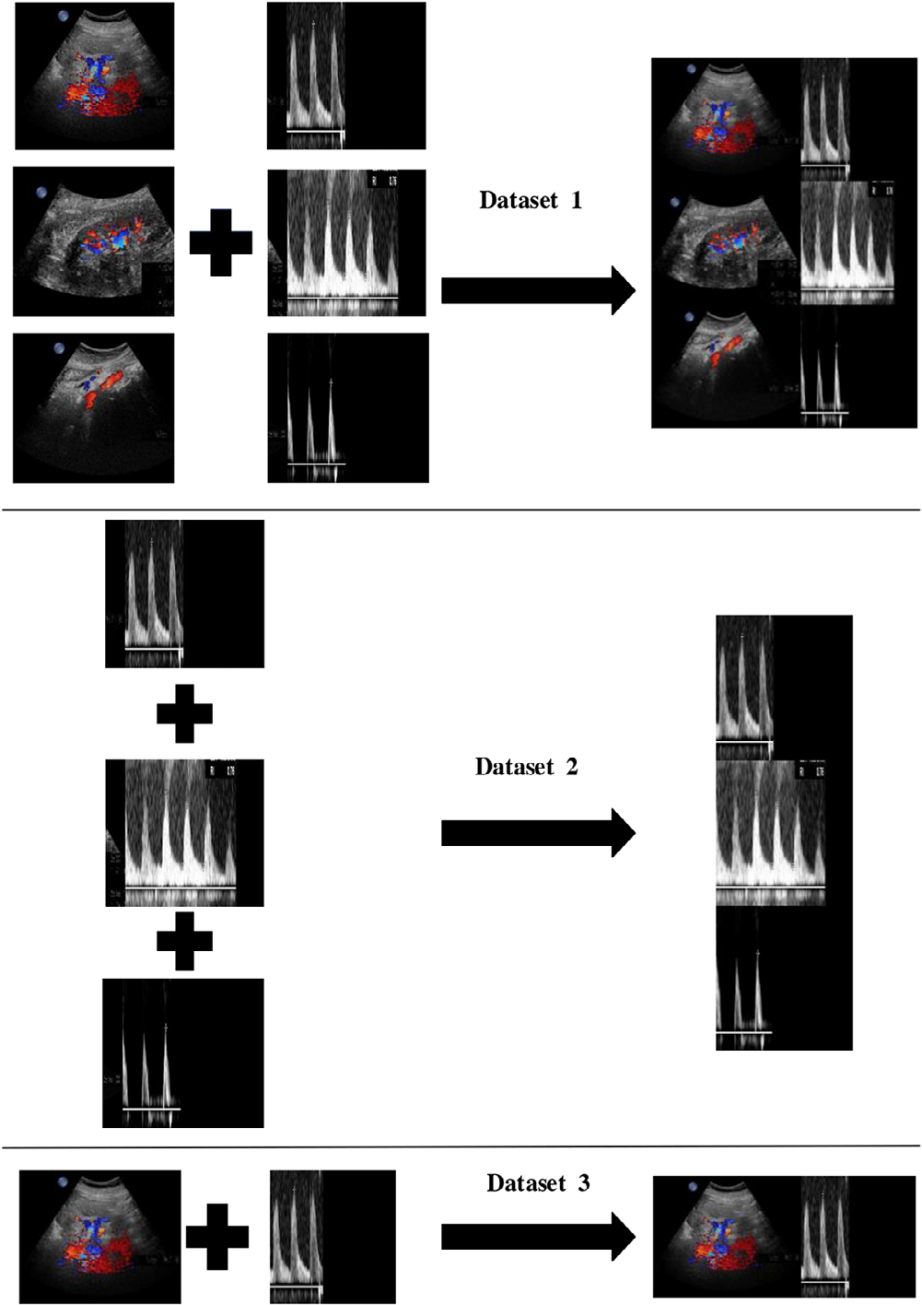
Schematic diagram of image fusion.

To further investigate the effectiveness of employing multimodal data for DL within the model, we constructed three distinct datasets during our research: a multimodal information dataset (Dataset 1), a dataset containing solely spectral region information (Dataset 2), and a dataset exclusively consisting of renal artery image information (Dataset 3). This study adopted the Holdout Validation method as our validation strategy. For both RAS and NRA patient categories, 80% of the data was allocated to the training set, 4% to the validation set, and 16% to the testing set for each category respectively. The schematic diagram of image fusion is shown in Figure 3.

Each model was tested using these three data sources for comparison, which allowed us to gauge their relative performance.

During the model training phase of our study, we primarily focused on addressing two core issues: preventing overfitting and tackling the problem of imbalanced samples. To prevent overfitting, we implemented an early stopping strategy. To streamline the model training process, we used ReduceLROnPlateau to dynamically adjust the learning rate. For the issue of sample imbalance, we considered class weights when defining the loss function to increase the model's focus on minority class samples. In the RAS diagnostic model training, cross‐entropy loss was used as our optimization target and Stochastic Gradient Descent (SGD) was the optimization algorithm.

For each model, the batch size was 8 and an initial learning rate was set to 0.01. In combination with ReduceLROnPlateau for dynamic adjustment of the learning rate, it was set to multiply the current learning rate by 0.1 if no improvements were seen over ten continuous training epochs. The RAS‐1 model consists of 269 layers in total. The output of the fully connected layer passes through a ReLU activation function and is mapped to a two‐dimensional output after a dropout layer. The RAS‐2 model has a total of 101 layers, maintaining the same fully‐connected layer configuration as RAS‐1. The image feature extraction part of the RAS‐3 model utilizes a Transform structure, with model depth being 24 layers, yet still maintains the same fully‐connected layer configuration as the previous two models. the class weights of cross‐entropy loss (CrossEntropyLoss) was set to 2.42 for the RAS and 1.70 for the RAN.

Models’ performance was compared and evaluated by calculating multiple metrics such as precision, recall, accuracy, F1 scores, ROC curves on the three datasets (Figure 3). Each model was run ten times, with the mean performance of the multiple runs taken as the final result. To demonstrate the stability and consistency of the models, we calculated the mean and standard deviation for each performance metric.

### 2.5 Model implementation and statistical analysis

In this study, image processing was conducted using Python 3.9.16 and the PyTorch DL library. During model construction, the “timm” library was utilized, specifically employing “resnest269e”, “resnetv2_101 × 1_bitm_in21k”, and “xcit_tiny_24_p8_384_dist” to assist in the model construction. The normally distributed variables were denoted as mean values with standard deviations, and categorical variables were formatted with counts and proportions. Ten trained results of every DL model were saved, and difference of predictive accuracies of different models were compared by the hypothesis testing. Independent two‐sample *t*‐test was used for variables obeying the normal distribution. The threshold of the *p*‐value was set to 0.05. Statistical analysis was conducted using IBM SPSS software (version 26.0, International Business Machines Corp., Chicago, IL, USA).

## 3 RESULTS

After filtering out patients with incomplete information through image preprocessing, we collected a total of 1485 patients' information for data set construction, among which 449 were diagnosed as the RAS and 1036 as the NRA (Table 1). For the RAS patients, there were 267 left and 272 right renal artery images in the RAS category. The NRA patients had more NRA (*N* = 1698 vs. 349), IRA (*N* = 1330 vs. 726), and AO images (*N* = 313 vs. 210) than the RAS patients. For NRA and IRA images, they were evenly distributed on the left and right orientations of RAS and NRA patients. For example, for the RAS patients, the NRA category contained 174 left and 175 right renal artery images. The IRA category included 349 left and 377 right IRA images.

One‐hundred seventeen patients with the gold standard results was classified as the test dataset, consisting of 277 RAS samples and 399 NRA samples respectively. For both RAS and NRA patient categories, 80% of the data was allocated to the training set, 4% to the validation set, and 16% to the testing set for each category respectively. The training set contained 3292 images (1365 RAS samples and 1927 NRA samples), the validation set included 169 images (70 RAS samples and 99 NRA samples), and the test set comprised 676 images (277 RAS samples and 399 NRA samples).

From Table 2, it could be found that there was no significant difference in accuracy between the Resnet and ResNeSt models (83.49 ± 0.45% vs. 81.84 ± 1.52%; *p* = 0.069). Conversely, the accuracy of XCiT (80.71 ± 2.23%) was lower than the ResNeSt and Resnet obviously (*p* < 0.001).

**TABLE 2:** Results of the three deep learning models trained on the multimodal dataset.

	Metrics	RAS‐1	RAS‐2	RAS‐3
**Training**	**Accuracy**	90.52% ± 3.36%	91.80% ± 2.29%	87.66% ± 1.51%
**Validation**	**Accuracy**	92.26% ± 1.22%	91.08% ± 0.92%	87.97% ± 1.95%
**Test**	**Accuracy**	83.49% ± 0.0045	81.84% ± 0.0152	80.71% ± 0.0223
	**Precision**	0.8189 ± 0.0300	0.7406 ± 0.0221	0.7414 ± 0.0203
	**Recall**	0.7697 ± 0.0370	0.8585 ± 0.0164	0.8152 ± 0.0157
	**F1 Score**	0.7924 ± 0.0066	0.7949 ± 0.0156	0.7759 ± 0.0104
	**ROC AUC**	0.9083 ± 0.0035	0.9078 ± 0.0102	0.8822 ± 0.0144
	***p*‐Value(compared to RAS‐1)**	–	0.069	*p* < 0.001

*Note*: The calculation of the *p*‐value of two models (RAS‐2 and RAS‐3) is in comparison with RAS‐1.

Abbreviations: DL, deep learning; RAS, renal artery stenosis.

In all three models, the accuracies on training and validation datasets exceeded 87%, and the RAS‐1′s training accuracy was 90.52%, the RAS‐2 was 91.80%. Three models's accuracies on the training and validation sets were generally higher than those on the test set. The accuracies hovered around 80% in the test set (e.g., RAS‐1 test accuracy = 83.49%, RAS‐2 test accuracy = 81.84%, and RAS‐3 test accuracy = 80.71%).

The RAS‐1 model had the highest accuracy (0.8349 ± 0.0045), precision (0.8189 ± 0.0300), and AUCs (0.9083 ± 0.0035). For the recall metric, the RAS‐2 model scored the highest (85.85 % ± 1.64%). RAS‐1 and RAS‐2 models shared similar F1 values (0.7924 ± 0.0066 vs. 0.7949 ± 0.0156). The RAS‐3 model outperformed RAS‐1 on the recall (0.8152 ± 0.0157 vs. 0.7697 ± 0.0370), but it differed significantly from the RAS‐1 and RAS‐2 models in other metrics.

In Table 3, we could find that for all three models (RAS‐1, RAS‐2, RAS‐3), the highest accuracy was achieved when using multimodal data, and the RAS‐1 achieving the highest accuracy overall (83.49% ± 0.45%). When comparing the performance across different datasets for each model, a clear pattern could be seen. For example, the RAS‐1′s accuracy is the best with multimodal data (83.49% ± 0.45%), followed by single spectral waveform data (81.18% ± 1.46%), and then single anatomical site data (80.89% ± 3.62%).

**TABLE 3:** Comparison of three deep learning models trained on three different datasets.

Model	Dataset	Accuracy	Precision	Recall	F1 Score	ROC AUC
**RAS‐1**	**Multimodal Data**	0.8349 ± 0.0045	0.8189 ± 0.0300	0.7697 ± 0.0370	0.7924 ± 0.0066	0.9083 ± 0.0035
	**Spectral waveform Data**	0.8118 ± 0.0146	0.7766 ± 0.0328	0.7639 ± 0.0501	0.7684 ± 0.0202	0.8874 ± 0.0094
	**Single vascular area Data**	0.8089 ± 0.0362	0.7434 ± 0.0665	0.8332 ± 0.0311	0.7829 ± 0.0281	0.8932 ± 0.0327
**RAS‐2**	**Multimodal Data**	0.8184 ± 0.0152	0.7406 ± 0.0221	0.8585 ± 0.0164	0.7949 ± 0.0146	0.9078 ± 0.0102
	**Spectral waveform Data**	0.8148 ± 0.0095	0.7820 ± 0.0279	0.7639 ± 0.0496	0.7711 ± 0.0170	0.9035 ± 0.0045
	**Single vascular area Data**	0.8053 ± 0.0334	0.7986 ± 0.0695	0.7686 ± 0.0868	0.7717 ± 0.0203	0.9016 ± 0.0119
**RAS‐3**	**Multimodal Data**	0.8071 ± 0.0223	0.7414 ± 0.0203	0.8152 ± 0.0157	0.7759 ± 0.0104	0.8822 ± 0.0144
	**Spectral waveform Data**	0.7562 ± 0.0140	0.7636 ± 0.0553	0.6087 ± 0.1230	0.6656 ± 0.0523	0.8363 ± 0.0201
	**Single vascular area Data**	0.7515 ± 0.0324	0.6453 ± 0.0542	0.8736 ± 0.0451	0.7423 ± 0.0321	0.8648 ± 0.0367

Abbreviation: RAS, renal artery stenosis.

During the performance testing of the models with three different datasets, the models showed consistency across all datasets. In the single spectral dataset and the dataset containing only renal artery images, all metrics, including Accuracy, Precision, Recall, F1 Score, and ROC AUC, demonstrated that the RAS‐1 and RAS‐2 models had similar performances. Specifically, the accuracy of the RAS‐1 model on these two datasets was approximately 0.8118 ± 0.0146 and 0.8089 ± 0.0362, respectively, while the accuracy of the RAS‐2 model was approximately 0.8148 ± 0.0095 and 0.8053 ± 0.0334. Both models also exhibited similar performances in terms of Precision, Recall, F1 Score, and ROC AUC. However, the performance of the RAS‐3 model was inferior to that of the RAS‐1 and RAS‐2 models across all metrics, with an accuracy of approximately 0.7562 ± 0.0140 and 0.7515 ± 0.0324 on these two datasets, significantly lower than the other two models. The performance of RAS‐3 model in terms of Precision, Recall, F1 Score, and ROC AUC was also significantly lower than that of the RAS‐1 and RAS‐2 models.

In Table 4, it can be observed that among them, RAS‐2 has the lowest Missed Diagnosis Rate (0.1415 ± 0.0164), RAS‐1 model has the lowest Misdiagnosis Rate (0.1811 ± 0.0300) and the highest Specificity (0.8189 ± 0.0300), while the performance of RAS‐3 model is relatively poor. Comparing the three datasets for the same model, the models using the Multimodal dataset exhibit the best overall performance.

**TABLE 4:** Medical metrics results of three deep learning models on three different datasets.

Model	Dataset	Missed Diagnosis Rate (FNR)	Misdiagnosis Rate	Specificity
**RAS‐1**	**Multimodal**	0.2303 ± 0.0370	0.1811 ± 0.0300	0.8189 ± 0.0300
	**Single Spectral**	0.2361 ± 0.0501	0.2234 ± 0.0328	0.7766 ± 0.0328
	**Single Anatomical Site**	0.1668 ± 0.0311	0.2566 ± 0.0665	0.7434 ± 0.0665
**RAS‐2**	**Multimodal**	0.1415 ± 0.0164	0.2594 ± 0.0221	0.7406 ± 0.0221
	**Single Spectral**	0.2361 ± 0.0496	0.2180 ± 0.0279	0.7820 ± 0.0279
	**Single Anatomical Site**	0.2314 ± 0.0868	0.2014 ± 0.0695	0.7986 ± 0.0695
**RAS‐3**	**Multimodal**	0.1848 ± 0.0157	0.2586 ± 0.0203	0.7414 ± 0.0203
	**Single Spectral**	0.3913 ± 0.1230	0.2364 ± 0.0553	0.7636 ± 0.0553
	**Single Anatomical Site**	0.1264 ± 0.0451	0.3547 ± 0.0542	0.6453 ± 0.0542

Abbreviation: RAS, renal artery stenosis.

In Table 5, the accuracy of RAS‐1 (Model compared with Clinicians) is displayed as 83.49%, which indicates that noise has a certain impact on the model. It could be observed that when comparing both the model's predictions and the clinicians' diagnoses against the gold standard, the clinicians' diagnoses accuracy was higher (90.09%), whereas the model's prediction accuracy was 78.25% ± 1.62%. There was more similarity between the model's prediction accuracy and the clinicians' diagnoses (90.09% vs. 78.25%), suggesting that the DL model mimicked the clinicians' diagnoses to a certain extent.

**TABLE 5:** Comparison of predicted results of deep learning model with the renal artery angiography.

	DL Model compared with Gold Standard	Clinicians compared with Gold Standard	RAS‐1(Model compared with Clinicians)
**Accuracy**	0.7825 ± 0.0162	0.9009	0.8349 ± 0.0045
**Precision**	0.6414 ± 0.0438	0.8454	0.8189 ± 0.0300
**Recall**	0.7385 ± 0.0139	0.9855	0.7697 ± 0.0370
**F1 Score**	0.6866 ± 0.0316	0.9101	0.7924 ± 0.0066
**ROC AUC**	0.8468 ± 0.0170	–	0.9083 ± 0.0035

Abbreviations: DL, deep learning; RAS, renal artery stenosis.

## 4 DISCUSSION

In this study, we proposed and developed a DL‐based multimodal fusion model for RAS diagnosis. During the process of training, validation, and testing, performance of three models, RAS‐1, RAS‐2, and RAS‐3 with different feature extraction modules were compared. Additionally, to assess the influence of different categories of data on model's accuracy, every model was trained further on either single‐modal spectral waveform images or only renal artery images excluding the AO and IRA images. The result showed that the RAS‐1 model, built using the ResNeSt backbone for image feature extraction and utilizing multimodal data, provided the best auxiliary diagnosis accuracy for RAS (92.26% ± 1.22% on validation dataset, and 83.49% ± 0.45% on test dataset). Compared to models trained on single‐modal dataset or images from single anatomical site, accuracies of models on multimodal dataset were higher.

Our results indicated that training with multimodal data significantly outperformed situations where only single spectral images or renal artery images were utilized (Table 3). A noticeable decline in performance metrics such as accuracy, F1 score, and ROC AUC was observed when relying solely on renal artery images (Table 3). These observations suggest that multimodal data, combining different image information and clinical text, encapsulates more valuable information. When employed on training and validation sets, this multimodal approach can generate a more promising model compared to single‐modal data, thereby bolstering the model's predictive capabilities.

Furthermore, the evaluation depicted in Table 4 elucidates the nuanced performance of our three models, RAS‐1, RAS‐2, and RAS‐3 across different datasets. It is evident that RAS‐1 model displayed the lowest Misdiagnosis Rate (0.1811 ± 0.0300) and the highest Specificity (0.8189 ± 0.0300), indicating its robustness in correctly identifying healthy individuals and minimizing false positives. On the other hand, RAS‐2 model showcased the lowest Missed Diagnosis Rate (0.1415 ± 0.0164), which is essential in reducing false negatives and thereby improving the sensitivity of RAS diagnosis. However, the performance of RAS‐3 model was found to be subpar, especially in terms of the Missed Diagnosis Rate on the Single Spectral dataset (0.3913 ± 0.1230). Moreover, the comparison of the three datasets for the same model revealed that the models trained on the Multimodal dataset exhibited the best overall performance, reinforcing the premise that a fusion of diverse data modalities can significantly enhance the diagnostic accuracy of RAS. This inference aligns with our earlier findings where the utilization of multimodal data yielded higher accuracies compared to single‐modal datasets.

However, when utilizing single spectral waveform data and single anatomical site data as inputs, the accuracy of RAS‐1 (81.18 ± 1.46% and 80.89 ± 3.62%) and RAS‐2 (81.48 ± 0.95% and 80.53 ± 3.34%) remained around 80%, with the RAS‐3 (75.62 ± 1.40% and 75.15 ± 3.24%) model's accuracy around 75%. This indicates that single‐modal data, including spectral images and renal artery images, also contain valuable auxiliary information for RAS diagnosis.

When comparing the performance of the three models, the accuracies on the training and validation sets were consistently higher than those achieved on the test set. The RAS‐1 and RAS‐2 models performed similarly (*p* > 0.05) and outperformed the RAS‐3 model (*p* < 0.05). The superior performance of ResNeSt (RAS‐1) and ResNet (RAS‐2) models, which employ CNNs, illustrated their capacity to better discern spatial hierarchies inherent in image data. The XCiT‐based model (RAS‐3), implementing a Transformer structure, exhibited a slightly less optimal performance, which may be due to the relatively small size of our current dataset. However, a notable advantage of the XCiT model is its faster training speed than RAS‐1 and RAS‐2. Other performance metrics (Precision, Recall, F1, and AUC) across the RAS‐1, RAS‐2, and RAS‐3 models showed similar patterns. This suggests that when employed as backbone networks, ResNeSt and ResNet are capable of extracting more meaningful information from our ultrasound and spectral image data, thereby enhancing model performance.

Upon comparing the test set results between the RAS‐1 and RAS‐2 models, we noticed that RAS‐1′s accuracy outperformed the RAS‐2, and demonstrated greater stability. This could be due to the characteristics of the ResNeSt architecture, short for Residual Networks with Stochastic Depth. ResNeSt introduces a “split‐attention” operation that allows different network paths to focus on different feature‐map subsets, enabling the model to learn a broader array of features. Moreover, ResNeSt stochastically drops some residual blocks during training, preventing overfitting and enhancing the model's generalizability. In contrast, the traditional ResNet model employs a straightforward architecture in which all network paths operate on all feature maps, potentially limiting the diversity of features it can learn.

Compared to renal artery angiography results, the accuracy rate of clinical physicians was 90.09%, while the model's predictive accuracy was 78.25% (Table 5). A possible reason for this discrepancy might be the limitation of training sample size, leading to a lower accuracy rate compared to physicians. The expertise of clinical physicians, encompassing years of experience and an professional understanding of disease progression, is a factor that the model hard to learn. Besides, considering the dynamic characteristic of ultrasound scanning, the static CDS image may lose some information compared to the scanning video. The results of the experiment demonstrated the effectiveness of this model in aiding the diagnosis of renal artery disease. This model integrates the actual process, simulating the doctors' diagnosis of renal artery disease, and is able to assist doctors in diagnosing renal artery disease.

Despite our multimodal models show promising result in aiding RAS diagnosis, we acknowledge several limitations. At present, our dataset including 4137 instances divided across training, validation, and testing sets. The sample size needs to be further expanded. To improve the prediction efficiency, the renal artery scanning video can be collected to capture richer information. Clinical validation is currently limited, and additional clinical verification will be required in future to ascertain the model's effectiveness. Besides, new structure of DL model should be designed to improve models' performance, and learn senior physician's experience better.

## 5 CONCLUSION

This study explores the possibility of DL multimodal fusion technology on assisting RAS diagnosis based on CDS image, spectral waveform image, and individual information. The DL model achieved promising performance and can be improved further to offers physicians real‐time, reliable diagnostic assistance, enhancing both accuracy and efficiency.

## AUTHOR CONTRIBUTIONS

Xin Wang collected and analyzed the patient image data and drafted the manuscript. Yuqing Yang implemented the deep learning object detection models and drafted the manuscript. Sheng Cai and Hongyan Wang revised the manuscript. Jianchu Li and Yuqing Yang designed this study and revised the manuscript. All authors read and approved the final manuscript.

## CONFLICT OF INTEREST STATEMENT

The authors declare are no conflict of interest.

## ETHICS APPROVAL

This study was approved by the Institutional Review Board of the Peking Union Medical College Hospital (Ethical review number: JS‐3306). All methods were carried out according to the Declaration of Helsinki. All participants gave informed consent.

## Data Availability

Data are available upon reasonable request to the corresponding author.
